# Supplementary material for: Long spin lifetimes of charge carriers in rubrene crystals due to fast transient-localization motion
Source: Nat Commun. 2025 Aug 15;16:7605. doi: 10.1038/s41467-025-62830-7 (PMC12356851; doi:10.1038/s41467-025-62830-7)
Supplement: Supplementary file 1 — Supplementary Information [file 41467_2025_62830_MOESM1_ESM.pdf]

Supplementary Information:
Long spin lifetimes of charge carriers in rubrene crystals due to fast transient localization motion

Remington L. Carey†[1], Xinglong Ren*†[1], Ian E. Jacobs[1], Jan Elsner[2], Sam Schott[1], Elliot Goldberg[1], Zichen Wang[1], Jochen Blumberger[2], and Henning Sirringhaus*[1]

[1]Cavendish Laboratory, University of Cambridge, Cambridge CB3 0HE, United Kingdom
[2]Department of Physics and Astronomy and Thomas Young Centre, University College London, WC1E 6BT, London, United Kingdom

† These authors contributed equally.
* Correspondence: xr216@cam.ac.uk; hs220@cam.ac.uk

Supplementary Note 1 Other techniques for spin relaxation time extraction

In this work we measure spin relaxation times solely by field-induced ESR, as discussed in the main text. However, there are other ways to measure the spin relaxation time. For example, the spin relaxation time can be measured by transport experiments such as spin valves, or by optical methods such as polarization sensitive spectroscopy. Each technique has its own advantages and limitations, which we discuss briefly here.

A spin valve is a device that uses the spin of electrons to control electrical resistance. Its key advantage is that the spin diffusion length, which is critical for device applications, can be directly extracted from the length (or thickness) dependence of the spin valve signal. However, extraction of spin relaxation time requires a separate measurement of the diffusion coefficient (or mobility). The spin valve was the first reported organic spintronic device, but local spin valves, where spin injection and detection occur between the same two electrodes, have known issues. The organic spin valve community has recognized that some spin-valve signals observed in organic local spin valves do not come from spin transport through organic molecules, but instead from artifacts such as the tunneling magnetoresistance effect.[1, 2, 3, 4] Non-local spin valve measurements can exclude most of the artifacts associated with local spin valve measurements and have provided reliable values in materials like graphene[5] and GaAs[6]. But unfortunately, it has not been achieved in organic semiconductors yet. Another method to extract the spin relaxation time from a spin valve is the Hanle effect measurement, where the spin valve signal is measured as a function of the out-of-plane magnetic field. However, so far no unambiguous Hanle effect has been observed in organic spin valves either.

Spin transport and relaxation can also be measured optically using polarization sensitive techniques like magneto-optical Kerr or Faraday rotation, where the change in polarization is related to the magnetization of the measured material. Usually materials with stronger spin-orbit coupling exhibit stronger rotation. The spin relaxation time can be extracted from the time-resolved rotation trace or from the Hanle depolarization due to the magnetic field. This method does not require the fabrication of the whole electronic device and has been widely used to measure spin relaxation times in many inorganic semiconductors such as GaAs[7] and ZnO[8]. However, no optical spin relaxation measurement has been reported in organic semiconductors such as rubrene, probably due to their weak spin-orbit coupling.

Although spin relaxation time can be measured by different techniques, when the sample quality is high enough and the measurements are properly performed, the results should be comparable. For example, spin relaxation times on the order of 1-10 ns have been observed in graphene by both non-local spin valves[5] and ESR[9], while for GaAs both non-local spin valves[6] and magneto-optical Kerr effect[7] yield values of 10-100 ns.

Supplementary Note 2 Electrochemical reaction at high gate voltages

Though the double-integrated intensity of the spectra (and therefore number of injected spins) grew with the magnitude of bias voltage applied (shown in Figure 1(b) in the main text), we do not report data for gate voltages $V_g < -1.5$ V. This because we observed a second ESR resonance signal develop in preliminary samples when measuring at high gate voltages. One such example is shown in Supplementary Figure 1. There, two curves are shown: the blue one corresponds to a spectrum taken immediately after applying the bias voltage of -2.0 V, while the red one was taken 70 minutes later. In both curves, the extremely narrow resonance of rubrene is observable near 3341 G. However, a second, much broader signal is observable as well, and grows in both intensity and linewidth with time. Because the rubrene signal was so much more narrow than the broader signal, we were unable to fit both spectra simultaneously. Further, because we ascribe this to an electrochemical process involving the rubrene and the ions in the ionic liquid and/or other electrochemical species, we believed such a process could interfere with spin lifetimes. Thus, we only recorded measurements in which we were sure no such reaction took place.

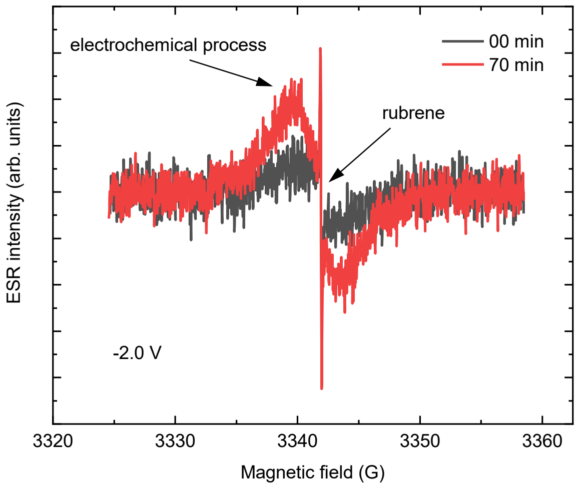

Supplementary Figure 1: **Appearance of an electrochemical signal.** An electrochemical signal appeared superimposed on the rubrene signal when devices were held at large gate voltages (more extreme than approximately -1.5 V) for too long. For this reason, we do not report data taken at such large gate voltages.

We have performed cyclic voltammetry (CV) measurements on a metal-ionic liquid-metal structure using the same ionic liquid [BMP][TFSI] as used in our original experiments. Our CV results suggest that the ionic liquid is stable within the voltage range used in our ESR measurements. As shown in Supplementary Figure 2, after 2 V, the current starts to increase rapidly with increasing voltage. For the 2.5 V scan, a smaller peak in the reverse scan can be observed at about 1.3 V, indicating some electrochemical reactions, and this peak becomes more obvious in the 3 V scan. We also did a stress experiment by holding the voltage at 1.5 V for 30 min (typical time for a single ESR scan), and found no obvious change in the CV curves before and after stress. We thus conclude that there is no stability issue if the gate voltage is below 1.5 V.

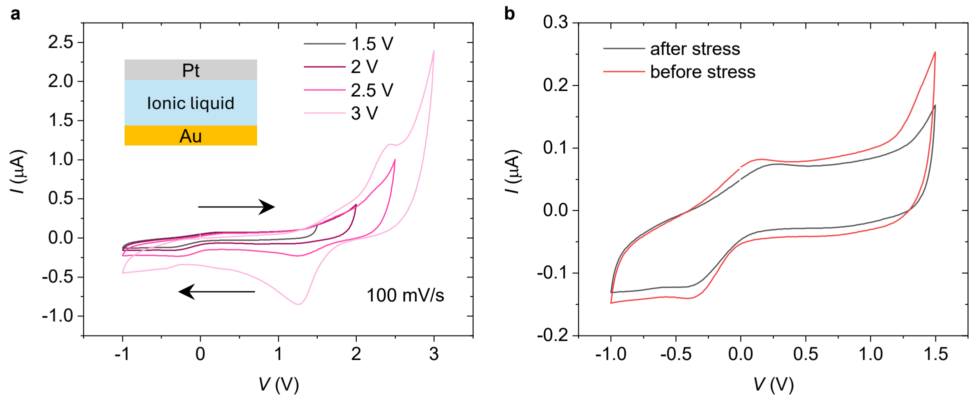

Supplementary Figure 2: **Cyclic voltammetry measurements of the ionic liquid [BMP][TFSI] at room temperature.** (a) CV curves measured at different voltage ranges. Inset shows the device structure. (b) CV curves taken before and after biasing the device at 1.5 V for 30 min. For CV measurements, the voltage is applied to the Au electrode, so the sign is opposite to the sign of the gate voltage for EDLTs.

Supplementary Note 3 Extraction of activation energy E_A from ion-gel devices.

In the main text, we note that the Redfield equations for relaxation in the regime of motional narrowing are

$$\begin{aligned} \frac{1}{T_1} &= \gamma_e^2\left(\overline{\mathscr{B}_x^2}+\overline{\mathscr{B}_y^2}\right)\frac{\tau_c}{1+\omega_L^2\tau_c^2} \\ \frac{1}{T_2} &= \frac{1}{2T_1}+\gamma_e^2\overline{\mathscr{B}_z^2}\tau_c \\ \frac{1}{T_2^*} &\equiv \frac{1}{T_2}-\frac{1}{2T_1}=\gamma_e^2\overline{\mathscr{B}_z^2}\tau_c, \end{aligned} \qquad \text{(Supplementary Equation 1)}$$

The correlation time τ_c is in general temperature dependent, however the functional form of this dependence is determined by the processes that allow spins to sample different local fields, e.g. charge motion. To determine $\tau_c(T)$, we recall that it can be interpreted as the inverse hopping frequency ν in the regime of motional narrowing (which is the regime of relaxation in the data from approximately 15 to 270 K) [10]. The Einstein relation for drift then relates mobility and hopping frequency via $\mu = q\overline{R}^2\nu/k_BT$, where $\overline{R}$ is the average hopping distance. Further, charge motion in ion-gel-gated transistors is thermally activated, following specifically the Arrhenius expression $\propto \exp[-E_A/k_BT]$ [11, 12]. From these two relations, we find the temperature dependence of the hopping frequency and, by its inverse, the correlation time:

$$\nu(T) \propto T\exp\left[\frac{-E_A}{k_BT}\right]. \qquad \text{(Supplementary Equation 2)}$$

For most temperatures, this provides a reasonable estimate of T_2^* as a function of temperature via Supplementary Equation 1.

Supplementary Equation 2 suggests that the effective motion frequency for motional narrowing always decreases with decreasing temperature. In ion-gel-gated rubrene, however, this is not valid at low temperatures. To justify the functional form of motion frequency shown in Equation 2, we define 2 characteristic length scales: the inter-ion distance (L_i) and the charge localization length (L_c). L_i distinguishes the local, transient localization motion and the long-range, thermally activated transport. At low temperatures where thermal activation is not enough for a charge carrier to leave its local region, L_c simply equals L_i and becomes temperature independent (L_c should increase with decreasing temperature according to the transient localization framework, but it is already greater than L_i at room temperature). Consequently, the effective motion frequency for motional narrowing is also temperature independent, because the charge carrier experiences the same hyperfine fields at different temperatures. The hypothesis that L_c must be constrained by L_i at low temperatures is also consistent with the observation that in the FI-ESR measurements on the FET devices of Figure 3c we observe longer spin relaxation times, already at 200 K, than in the ion-gel gated devices. If the charge carrier can be thermally activated to leave its local region, then L_c becomes larger than L_i, and the effective motion frequency for motional narrowing depends on temperature (also thermally activated). Its functional form becomes the sum of a thermally activated term (contribution from thermally activated transport) and a constant term C (contribution from local transient localization motion), with the former

one dominating the temperature dependence at high temperature. To plot the resulting T_2^* against the data, we absorbed γ_e and $\overline{\mathscr{B}_{\text{rms}}}$ into the constants of proportionality (A). We therefore write

$$T_2^* = A \times T \exp\left[\frac{-E_A}{k_B T}\right] + C. \qquad \text{(Supplementary Equation 3)}$$

Supplementary Figure 3 shows fits of the experimental T_2^* data in Figure 2 in the main text according to Supplementary Equation 3 (Equation 2 in the main text) using a temperature-activated motion frequency with varying activation energies (30, 40, and 50 meV). Supplementary Figure 4 shows relaxation times vs. temperature for an additional device held at -1.0 V. We again see single-microsecond-long relaxation times across the range of temperatures recorded, as well as a near-monotonic increase in relaxation times from 20 K to 260 K. Supplementary Figure 4 shows the corresponding fits to the T_2^* data at -1.0V.

The best fits are obtained for an activation energy E_A = 40 meV. For the -1.5 (main text), -0.4 (main text), and -1.0 V (supplemental material) curves, the values of best fit for the other two parameters are A = 17, 26, and 73 ns/K, and C = 0.8, 1.8, and 0.9 μs, respectively. At higher gate voltages, the constant C becomes smaller. As mentioned above, C is related to the length scale of the fast, transient localization motion. We believe it can be considered as an indicator for the density of counter-ions (or the inter-ion distance L_i). If the ion density is small (L_i is large), the local motional narrowing will be more efficient because the charge carriers are more delocalized, which should lead to larger spin lifetimes (and larger C) at low temperatures. Another potential effect of gate voltage is that charge carriers will be less strongly attracted and confined to the interface at lower gate voltages and will therefore undergo faster local motion due to the reduced influence of potential corrugation. Both factors would lead to a smaller C at a higher gate voltage.

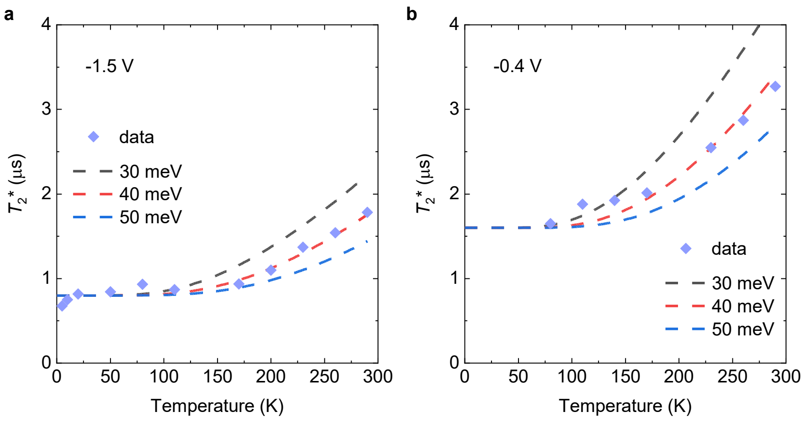

Supplementary Figure 3: **Determination of E_A.** Fits to the T_2^* data as a function of temperature using varying activation energies via Supplementary Equation 3. The fits are shown for gate voltages of -1.5V (a) and -0.4V (b)

.

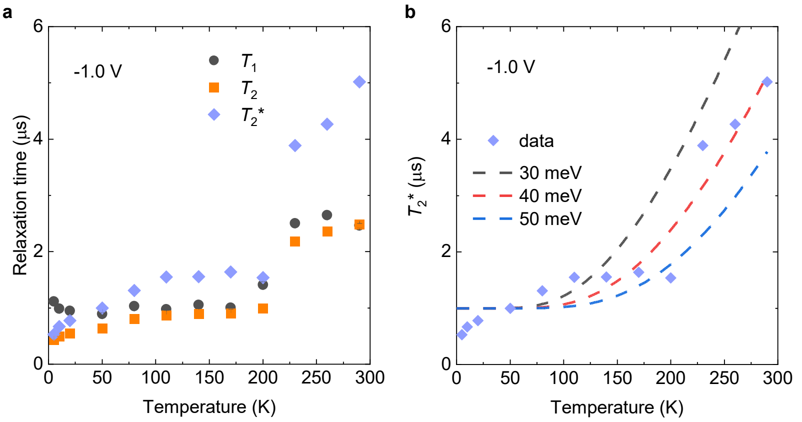

Supplementary Figure 4: **FI-ESR data for a third ion-gated device.** (a) Relaxation times in a device measured at -1.0 V; spin lifetimes are again microsecond-long. (b) The corresponding fits to T_2^* using varying activation energies via Supplementary Equation 3. Error bars represent standard deviations.

Supplementary Note 4 Redfield fits of spin relaxation times

In Supplementary Figure 5, we show full Redfield fits for T_1, T_2, and T_2* at a relatively low charge carrier density (V_g = -0.4 V). The fitting protocols can be found on Github (https://github.com/OE-FET/Rubrene-ESR-Fitting). This carrier density is below that in which we observe a decrease in T_2 with increasing carrier density (Figure 5), indicating that relaxation is still dominated by hyperfine interactions rather than dipolar coupling between carriers. At higher carrier density well into the strong dipolar coupling regime (V_g = -1.5 V) we are no longer able to consistently fit T_1, T_2, and T_2^* using a single $\overline{\mathscr{B}_\mathrm{rms}}$ value (compare Supplementary Figures 6a-c and 6e-g), and instead require an anisotropic fluctuating field ($\overline{\mathscr{B}_{xy}} \neq \overline{\mathscr{B}_z}$). In these anisotropic fits (Supplementary Figure 6e-h) we also obtain nearly an order of magnitude larger fluctuating fields as compared to the low carrier density case shown in Supplementary Figure 5. This in turn leads to a τ_c that is significantly shorter than $1/\omega_\mathrm{L}$, meaning that charge motion remains in the extreme narrowing regime at all measured temperatures. In this case, we are unable to uniquely determine the hopping rate $1/\tau_\mathrm{c}$ because all three relaxation times, T_1, T_2, and T_2^*, become invariant to changes in τ_c so long as $\overline{\mathscr{B}^2_\mathrm{rms}}\tau_\mathrm{c}$ remains constant. At best, we can instead estimate an upper bound on the correlation time, as indicated by the dashed line fit in Supplementary Figure 6h.

The larger magnitude of the fluctuating fields in this high carrier density sample result from the dipolar contribution to relaxation from neighboring charge carriers that are absent at lower carrier densities. The dipolar coupling model given in the main text predicts identical T_1 and T_2 values, which when interpreted through the Redfield equations imply $\overline{\mathscr{B}_{xy}} = \overline{\mathscr{B}_z}$. However, this model is derived under the assumptions of 3D random isotropic motion, while in our devices the spins are in reality confined to a nearly 2D plane normal to B_0. This reduction in dimensionality changes the lattice operators $F_k^{(2)}$, and is expected to result in different dipolar contributions to T_1 and T_2, leading to an anisotropic fluctuating field when treating these data with Redfield theory.[13] However, a quantitative description of this behaviour is beyond the scope of this work.

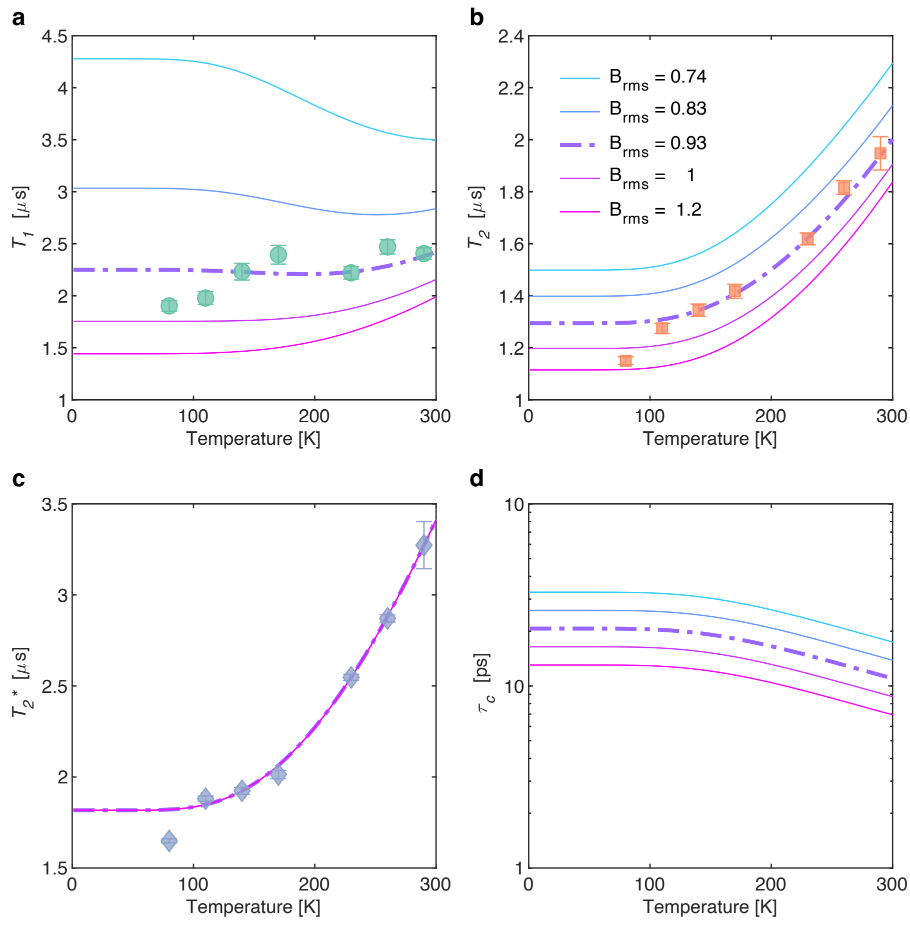

Supplementary Figure 5: **Redfield fits to ion-gel-gated Rubrene devices at $V_G = -0.4$ V.** Fits to (a) T_1, (b) T_2, and (c) T_2^* for various $\overline{\mathscr{B}_{\mathrm{rms}}}$ values, where the product $\overline{\mathscr{B}_{\mathrm{rms}}^2}\tau_{\mathrm{c}} \propto 1/T_2^*$ has been held constant. (d) Extracted τ_{c} for fits shown in (a-c). The variations in T_1 vs. T in (a) are not systematic, which suggests the fit error is dominated by experimental uncertainty that likely originates from the difference in the way T_1 and T_2 are measured. T_1 is obtained by fitting the evolution of the resonance line width and intensity as the microwave power is increased, and therefore is susceptible to error from fluctuations in microwave power or errors in cavity tuning. For example, if the sample position (which is difficult to precisely control when loading the sample into the microwave cavity) was shifted by 1 mm, there would be a 0.03 μs change in T_1 due to the change of microwave power. T_2, on the other hand, is derived only from the linewidth well below saturation, which is insensitive to fluctuation in these parameters. The T_2 data in (c) are therefore better described by the model. Error bars represent standard deviations.

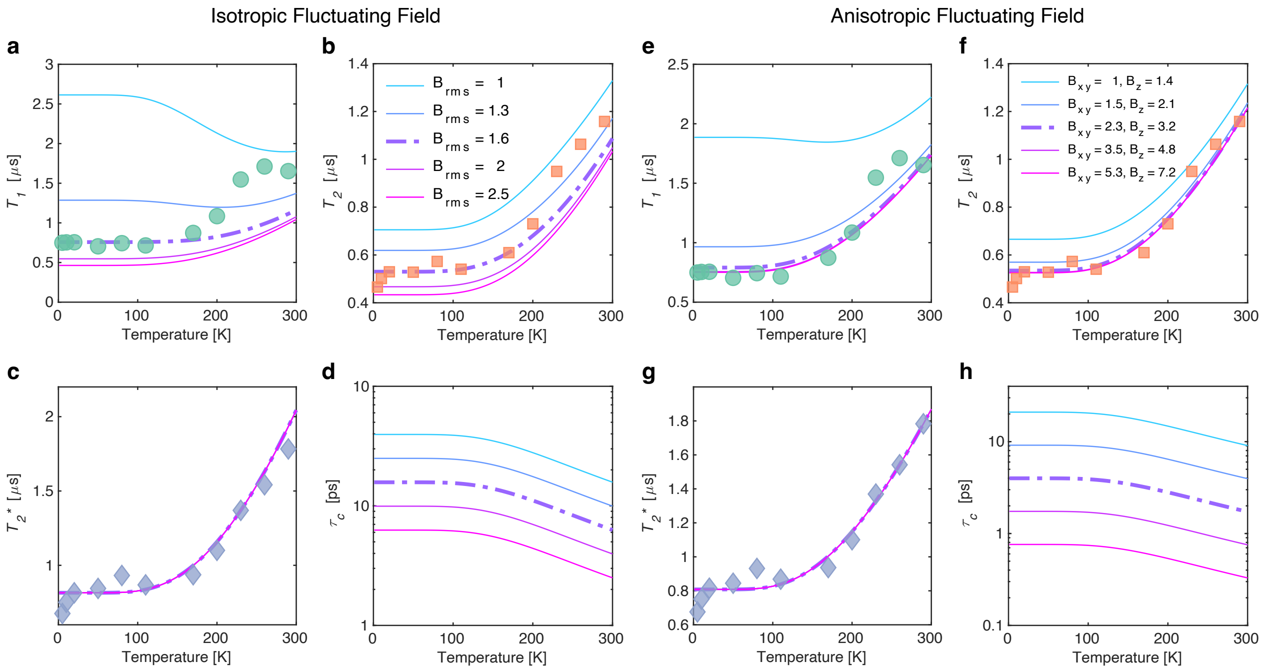

Supplementary Figure 6: **Redfield fits to an ion gel device gated into the strong dipolar coupling regime.** (a-d) Fits using an isotropic fluctuating field ($\overline{\mathscr{B}_{\rm rms}}$): (a) T_1, (b) T_2, and (c) T_2^* for various $\overline{\mathscr{B}_{\rm rms}}$ values, where the product $\overline{\mathscr{B}_{\rm rms}^2}\tau_{\rm c} \propto 1/T_2^*$ has been held constant. (d) Extracted $\tau_{\rm c}$ for fits shown in (a-c). (e-h) Fits using an anisotropic, axially symmetric fluctuating field ($\overline{\mathscr{B}_x} = \overline{\mathscr{B}_y} \neq \overline{\mathscr{B}_z}$): (e) T_1, (f) T_2, and (g) T_2^* for various $\overline{\mathscr{B}_{xy}}$ and $\overline{\mathscr{B}_z}$ values, where the product $\overline{\mathscr{B}_z^2}\tau_{\rm c} \propto 1/T_2^*$ and the ratio of $\overline{\mathscr{B}_{xy}}$ to $\overline{\mathscr{B}_z}$ have been held constant. (h) Extracted $\tau_{\rm c}$ for fits shown in (e-g).

Supplementary Note 5 Measurements on an Electric Double-Layer Transistor (EDLT)

As mentioned in the main text, we excluded mobility as the cause of the non-monotonic gate-voltage dependence of T_2 before developing our model based on the spin-spin interaction. We did so by performing ESR measurements on a rubrene single crystal EDLT (Supplementary Figure 7, inset) fabricated under similar conditions than the capacitor devices: thin rubrene crystals were transferred onto pre-fabricated Au source/drain electrodes, followed by lamination of the ion gel film and the Pt gate. Thermal stress effects limited these measurements to room temperature, but in the EDLT we also observed a peak in T_2 at $V_g = -0.3$ V and very similar carrier concentration of 2.5×10^{12} cm^{-2} (Supplementary Figure 7a). However, the corresponding EDLT transfer characteristics (Supplementary Figure 7b) do not show a peak in the current I_{sd} or the extracted mobility around this value. We note that a peak in conductance and mobility has been observed in similar EDLT devices previously,[14] but only at significantly higher carrier concentrations greater than 10^{13} cm^{-2}. The transfer curve in Supplementary Figure 7b shows hysteresis, which is likely due to the slow motion of ions instead of any unstable behaviour. If we further decrease the gate voltage scan rate, the hysteresis would be reduced. The reliability of our ESR results is not affected by the hysteresis in the transfer curve, because we didn't take ESR scans while recording transfer curves. Instead, before taking ESR scans, we first set the gate voltage and hold it for several minutes to make sure that the double layers have formed and the device is at equilibrium.

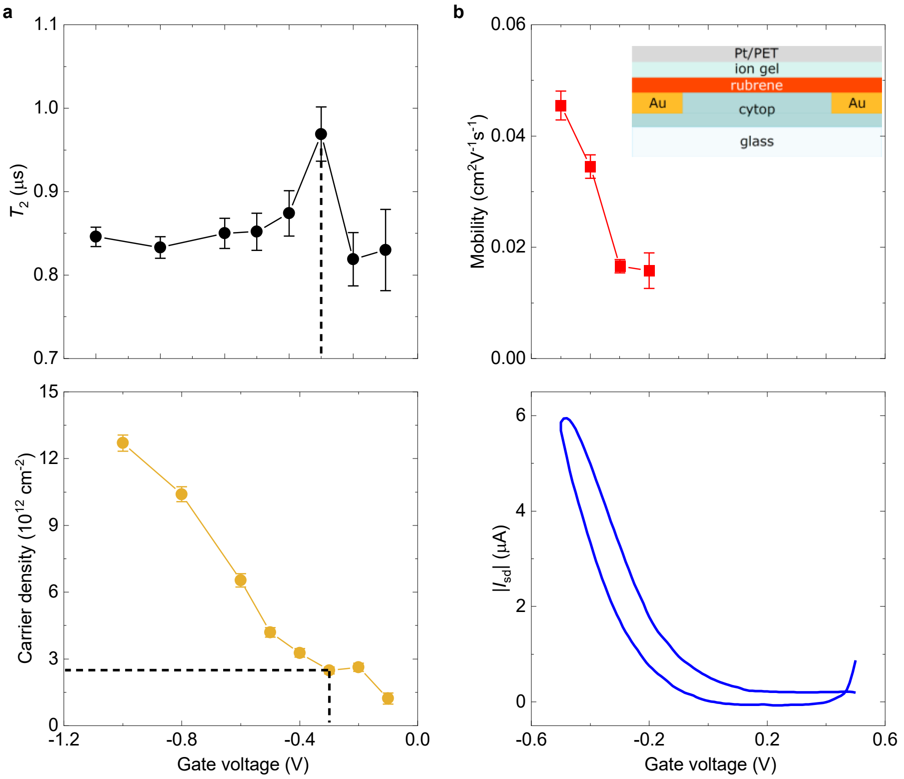

Supplementary Figure 7: **Measurements on a rubrene EDLT.** (a) The extracted spin concentration (bottom) and T_2 (top) vs. applied gate voltage; a peak in T_2 was observed at $V_g = -0.3$ V. (b) The transfer curve (bottom, $V_{sd} = -0.1$ V) and corresponding mobilities (top) of the same device before ESR scans were taken. Mobilities are only reported at gate voltages for which we estimated the number of spins via ESR. Inset: a schematic of the EDLT architecture. Error bars represent standard deviations.

We extracted the mobility of the EDLT from its transfer characteristics via

$$\mu = \frac{L}{W} \frac{I_{sd}}{q N_{spin} V_{sd}} \qquad \text{(Supplementary Equation 4)}$$

(where L and W are the channel length and width, respectively).[15]

Supplementary Note 6 Device structure of conventional rubrene FETs

The most well-known feature of transient localization in rubrene FETs is probably the band-like temperature dependence of mobility (i.e., increasing mobility with decreasing temperature). Most experimental observations of band-like transport reported so far are based on the so-called "air-gap transistor" geometry with a high quality, pristine semiconductor/dielectric interface [16, 17, 18, 19, 20]. However, the very small capacitance of the air-gap prevents us from applying this device geometry in FI-ESR experiments. For example, for a commonly used 5 µm air-gap with a specific capacitance of 0.18 nF/cm^2 [19], the charge density at 100 V is only around 10^{11} cm^{-2}, which means a channel area of 1 cm^2 is needed to achieve the detection limit (10^{11} spins). This channel area is not realistic, because the dimensions of the ESR substrate is only 0.3 cm $\times$ 4 cm and obviously the actual channel area is much smaller than the substrate. Therefore, a dielectric layer with higher capacitance is needed. However, for rubrene FETs with other dielectrics, the temperature dependence of mobility is usually thermally activated, so the choice of dielectric materials is quite limited.

It has been reported that band-like transport can be observed in Cytop-gated rubrene FETs [21, 22]. Cytop has a relatively small dielectric constant ($\sim$2.1) compared with SiO_2 ($\sim$3.9), so instead of using pure Cytop, we have decided to spin coat a thin layer of Cytop (40-45 nm) on top of 300 nm SiO_2 in order to maximize the capacitance while keeping the high quality Cytop/rubrene interface. We have successfully observed band-like temperature dependence of mobility in rubrene single crystal FETs fabricated using this method. As shown in Figure 3 and Supplementary Figure 8, regardless of the channel direction, both devices exhibit clear band-like feature (higher sheet conductivity and mobility at lower temperature). Note that since the transfer curve is not perfectly linear and the mobility value is gate-dependent, we use the effective mobility as suggested by Choi et al. [23] to compare the mobility values at different temperatures. The transient localization theory predicts a power-law temperature dependence of mobility ($\mu \propto T^{-n}$) in perfectly clean crystals, with the exact value of n depending on the degree of disorder. For example, a recent simulation work suggests n is in the range of 1.2 to 1.7 for perfect rubrene crystals[24] However, in real experiments, the situation is more complicated due to the unavoidable existence of extrinsic sources of disorder. Both the magnitude of mobility and the exponent n are usually smaller than theoretical values. $n > 1$ has only been reported in the best rubrene air-gap transistors thanks to the very clean rubrene/air interface (an example can be found in [19]). For cytop-gated rubrene transistors, n is usually smaller due to the increased extrinsic disorder. For example, values from 0.30 to 0.42 have been reported in the literature[21, 22]. Our data in Figure 4b has an exponent of 0.37, which is comparable to literature values.

The rigid nature of SiO_2/Cytop substrate, however, prevents electrical measurements at very low temperatures. Due to the mismatch of the thermal expansion coefficients, a tensile strain is applied to the rubrene crystal (rubrene has larger thermal expansion coefficients than SiO_2/Cytop) at low temperatures. Additionally, only those thin, flexible rubrene crystals (thickness < 1 µm) that are less mechanically robust can spontaneously adhere to the rigid SiO_2/Cytop substrate to form good contacts. As a result, thin rubrene crystals usually crack at low temperatures due to tension, and that is why our measurements on conventional FETs were performed over a limited temperature range.

On the other hand, for ion-gel-gated rubrene crystals, we choose to use thicker crystals ($\sim$100 µm thick) that are mechanically more robust so that our FI-ESR measurements can be performed at much lower temperatures down to 15 K. Thanks to the large capacitance of ion gel, only one crystal is needed to provide enough numbers of spins. The disadvantage of using thicker crystals is that thicker crystals cannot spontaneously adhere to rigid substrates, so a small drop of Ag paint is needed to affix the crystal and to serve as the electrical contact for the rubrene capacitor.

Supplementary Note 7 Additional data for a silicon dioxide/Cytop FET

As mentioned in the main text, we performed electrical and FI-ESR measurements on SiO_2 FETs to show that the behavior of relaxation times in a device operating in the regime of purely transient localization. The results of one device are presented in the main text. The results of a second device are shown in Supplementary Figure 8. This device has a lower mobility compared with the device shown in Figure 3 because its channel direction is closer to the low mobility direction of rubrene single crystal.

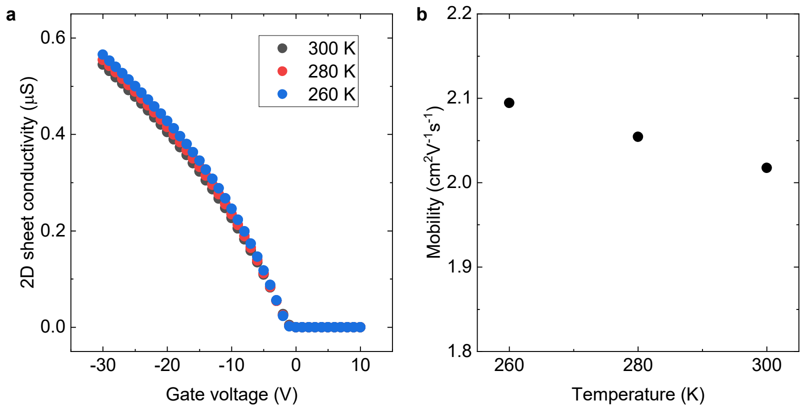

Supplementary Figure 8: **Charge transport data of a second conventional rubrene FET**. (a) Conductivity and (b) corresponding mobility data at 260, 280, and 300 K. The temperature-inhibited mobility confirms that charge transport in this device is dictated by transient localization.

Supplementary Note 8 Measurements of the displacement current

Measurements of the displacement current are taken by recording an I_g vs. V_g curve for different sweep rates, integrating the result, and dividing by the sweep rate.[15, 12] Though values of the displacement-current-extracted carrier concentration in Figure 5(a) (main text) diverge from those of ESR at higher gate voltages, they coincide at the peak in T_2. The divergence may be attributable to an overestimate by the displacement current method due to increased gate leakage at higher gate voltages, or to an underestimate of the ESR-extracted value due to anti-aligned spin pairs at high carrier concentrations.

Supplementary Note 9 Discussion on the Rashba-type spin-orbit interaction in rubrene

Rashba-type spin-orbit interactions usually occur in materials with broken inversion symmetry. The rubrene crystals used in our devices were in the orthorhombic phase with inversion symmetry (space group Cmca). Additionally, unlike materials with strong Rashba effects[25, 26], there is no Rashba-type band splitting feature in the rubrene band structure revealed by ARPES[27, 28]. Moreover, we can not find any experimental observation of strong bulk Rashba effect in materials consisting of only hydrogen and carbon in the literature, so there should be no bulk Rashba effect in rubrene.

In materials with inversion symmetry, the Rashba effect can still be observed if the inversion symmetry is broken by the electric field at the interface. The strength of the Rashba effect would depend on the electric field, so if the Rashba effect plays a significant role, we would observe very different spin lifetimes under different electric fields. To address this, we performed finite element analysis to estimate electric fields using the **Semiconductor Module in COMSOL Multiphysics**. Details of the simulations are described below.

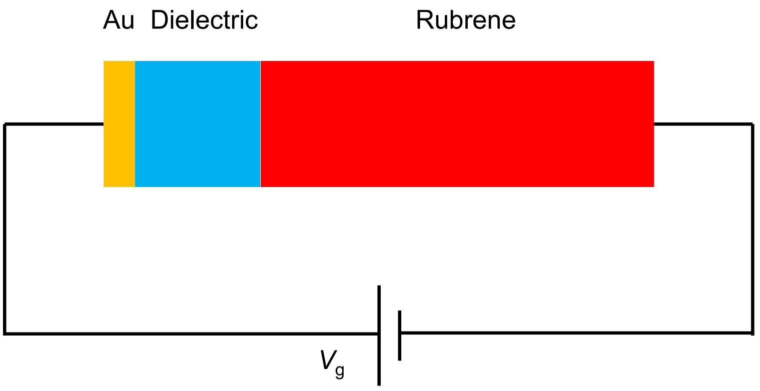

Supplementary Figure 9: **The gate-dielectric-semiconductor structure used for COMSOL simulations.**

(1) The device is a gate-dielectric-semiconductor capacitor as shown in Supplementary Figure 9. A gate voltage is applied to the Au gate, and the Poisson equation is solved for different gate voltages.

(2) The following parameters commonly reported in the literature are used for the semiconductor (rubrene) layer: $N_C = N_V = 1.46*10^{21}$ cm^{-3}, $E_g = 2.2$ eV, $E_C = 3.2$ eV, $E_V = 5.4$ eV, and $\varepsilon_{sc} = 3\varepsilon_0$ (N_C and N_V are the effective density of states in the conduction band (LUMO) and valence band (HOMO), E_g is the band gap, E_C and E_V are the LUMO and HOMO levels, ε_{sc} is the permittivity of the semiconductor, and ε_0 is the vacuum permittivity). The p-type doping level is set to be 10^{15} cm^{-3} throughout the entire layer, as suggested by a recent paper[29]. In the simulation, we set the thickness of rubrene to be 100 μm, but the results indicate that carrier accumulation primarily occurs within the first 1-2 nm from the rubrene/dielectric interface, as expected in a field-effect device.

(3) The left boundary of the rubrene layer (Au-dielectric stack) is modeled by the "thin insulator gate" boundary condition in COMSOL. The workfunction of Au is 5.1 eV. The following permittivity (ε_i) and thickness (t_i) values are used for the dielectric layer: for the cytop dielectric, $\varepsilon_i = 2.1\varepsilon_0$, and $t_i = 200$ nm (specific capacitance 9.3 nF cm^{-2}); for the ionic liquid dielectric, $\varepsilon_i = 10\varepsilon_0$, and $t_i = 2$ nm (specific capacitance 4.4 μF cm^{-2}).

(4) The right boundary of the rubrene layer is modeled by the "metal contact" boundary condition in COMSOL. The voltage is set to be 0 V and the contact is ideal Ohmic.

(5) Both Fermi-Dirac statistics and Boltzmann statistics are used in the simulations.

The electric fields at the rubrene/dielectric interface as a function of gate voltage for both a cytop-gated conventional FET and an ionic-gated EDLT are shown in Supplementary Figure 10. The highest electric fields for the FET and for the EDLT are around 0.1 V/nm and 0.25 V/nm, which are on the same order as what we would expect from their capacitance values. For the conventional FET, Fermi-Dirac statistics and Boltzmann statistics give nearly identical curves. For the ion-gated EDLT, the 2 curves match very well at low gate voltages but start to diverge at high gate voltages, because the carrier density becomes high enough that the semiconductor is no longer non-degenerate (in the simulation, the carrier density at -1 V is $2.75*10^{13}$ cm^{-2}, on the order of 0.1 charge/molecule). A discussion on whether the system is degenerate or not can be found in Supplementary Note 10. But importantly, for the EDLT, within the carrier density range used in our experiment, the electric field can be modulated over one order of magnitude, regardless of the carrier statistics. Since the Rashba coupling coefficient is proportional to the electric field, the strength of the Rashba effect should also change over one order of magnitude.

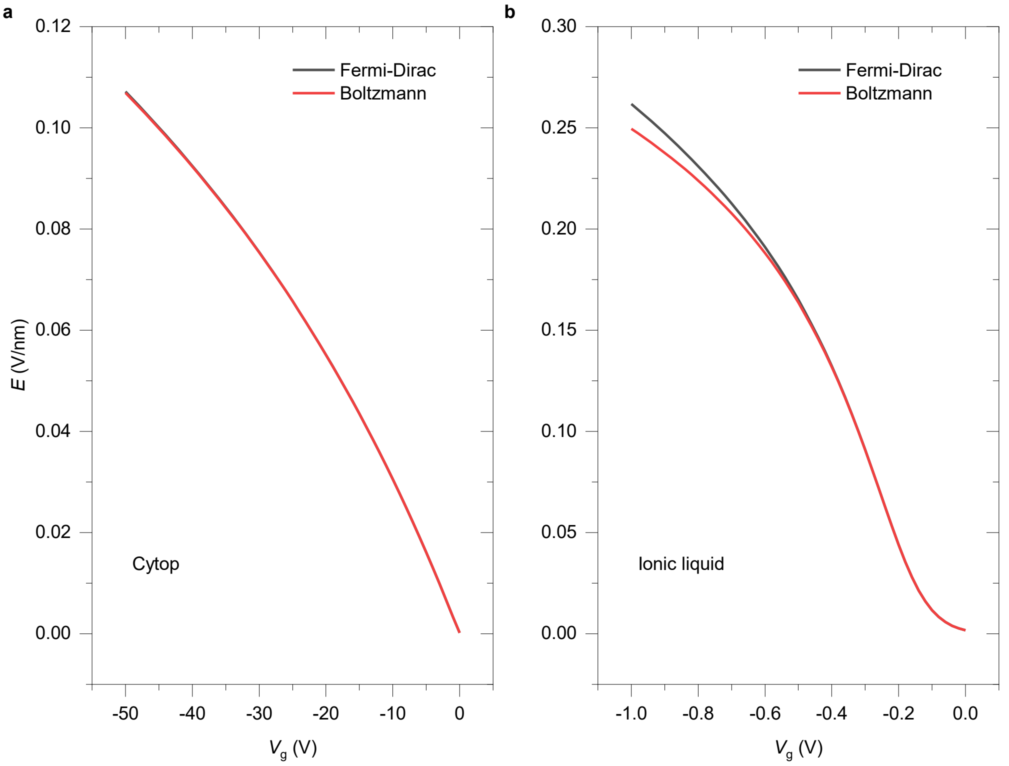

Supplementary Figure 10: **Electric field at the rubrene/dielectric interface from COMSOL simulations.** Both the cytop-gated FET (a) and the ion-gated EDLT (b) are simulated. Results for both Fermi-Dirac statistics and Boltzmann statistics at 300 K are displayed.

Next, we estimate what should we expect in our ion-gated rubrene EDLTs if the Rashba effect is playing a dominant role in spin relaxation. The Rashba coupling coefficient is inversely proportional to the square root of the spin relaxation time[30], so a 10-fold increase in the Rashba coupling coefficient would lead to a

100-fold reduction of the spin relaxation time. However, this is not supported by our existing results: the spin lifetimes are nearly independent of gate voltage (electric field), as shown in Figure 5. In addition to our results, the gate voltage dependence of ESR linewidth reported in ref. [11] in the main text is also very weak.

Previously, by performing ESR measurements on deuterated rubrene, our group demonstrated that spin-orbit coupling is much weaker than hyperfine interaction in isolated rubrene molecules in solution (ref. [43] in the main text). In this work, we do not observe any signature of spin-orbit interaction in rubrene crystals either. Therefore, we strongly believe that spin-orbit coupling does not play an important role in spin relaxation in rubrene.

Supplementary Note 10 Discussion on the validity of the Einstein relation

As shown by Blom et al.[31], the classical Einstein relation can be valid even in disordered polymer semiconductors when the influence of deep traps is eliminated. Moreover, work done previously in our group has shown that the classical Einstein relation fits the ESR results well in disordered polymeric semiconductors measured under similar conditions[10]. Rubrene has a much lower level of deep trap states compared to the disordered polymers used in our past work and, indeed, than those considered by Blom et al., meaning that our rubrene single-crystal devices should also be sufficiently at equilibrium.

It is also important to notice that the classical Einstein relation is only valid when the semiconductor is non-degenerate and Fermi-Dirac statistics can be approximated by Boltzmann statistics. The key parameter is the Fermi level position as a function of carrier density, and we have estimated it using the method described below.

According to the ARPES measurements[27], the HOMO bandwidth of rubrene is 0.4 eV. Still, we need to make some assumptions about the density of states (DOS) profile due to the lack of experimental data. A simulation by Troisi[32] suggests that the DOS for a 2D rubrene lattice is weakly dependent on energy, so we assume a constant 2D DOS for our calculations. Considering that the 2D molecular density of rubrene is $2*10^{14}$ cm^{-2} ($4*10^{14}$ cm^{-2} carriers are needed to fully empty the band), the magnitude of DOS is 10^{15} cm^{-2} eV^{-1}. While assuming a constant DOS might be somewhat simplistic as the real DOS should exhibit some energy dependence, we believe that it should provide reasonable order-of-magnitude information on the Fermi level shift.

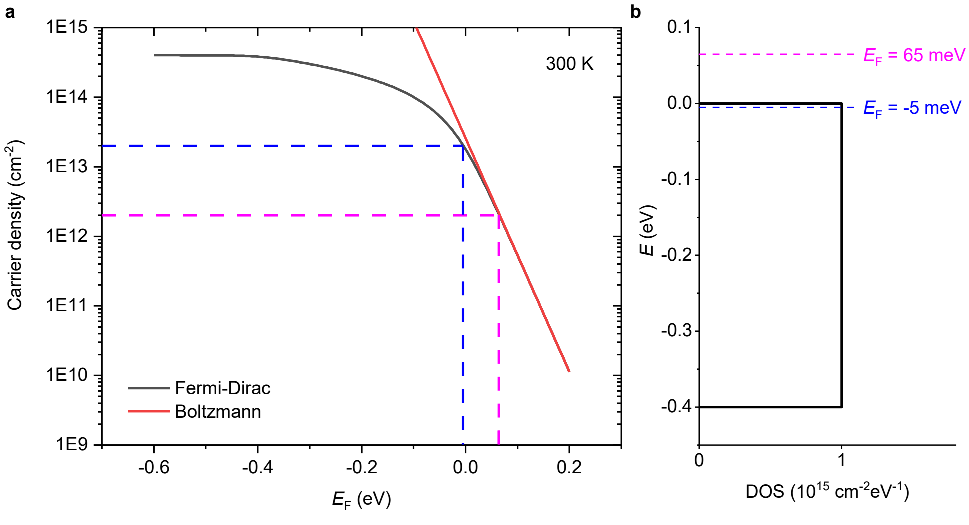

Supplementary Figure 11: **Fermi level shifted after doping at 300 K.** (a) Calculated relationship between carrier density and Fermi level at 300 K. Dashed lines represent carrier densities of 0.1 charge/molecule (blue, $2*10^{13}$ cm^{-2}) and 0.01 charge/molecule (pink, $2*10^{12}$ cm^{-2}) respectively. (b) The corresponding positions of Fermi level with reference to the top of the HOMO band.

The calculated relationship between carrier density and Fermi level at 300 K is shown in Supplementary Figure 11. We highlight 2 representative carrier densities: 0.01 charge/molecule ($2*10^{12}$ cm^{-2}) and 0.1 charge/molecule ($2*10^{13}$ cm^{-2}). At 0.01 charge/molecule, the Fermi level lies 65 meV above the top of the HOMO band, which is much larger than the thermal energy (26 meV). Consequently, Fermi-Dirac statistics can be reasonably approximated by Boltzmann statistics (the difference is about 5%), and the semiconductor can be treated as non-degenerate. At 0.1 charge/molecule, however, the Fermi level is 5 meV below the top of the HOMO band. In this case, the system is degenerate, and the Boltzmann approximation is not valid.

These results suggest that our conventional FET devices (maximum carrier density $2.8*10^{12}$ cm^{-2}) can be considered non-degenerate, while there is a crossover from non-degenerate to degenerate in ion-gated EDLT devices (maximum carrier density over 10^{13} cm^{-2}).

We would like to emphasize that in our manuscript **the classical Einstein relation is only used to estimate diffusivity near the "T_2 peak"** in Figure 5, which corresponds to carrier densities of 2 to $3*10^{12}$ cm^{-2} (approximately 0.01 charge/molecule). As shown above, in this regime, the system can still be considered non-degenerate, and Fermi-Dirac statistics can be reasonably approximated by Boltzmann statistics. Therefore, the classical Einstein relation remains valid near the "T_2 peak". At carrier densities over 10^{13} cm^{-2}, the system becomes degenerate, and the classical Einstein relation needs to be replaced by the so-called generalized Einstein relation, but in our manuscript we **did not** apply the classical Einstein relation in this degenerate regime.

Another evidence for the non-degenerate nature of rubrene near the "T_2 peak" is the Curie-like spin susceptibility from ESR measurements. If the system were degenerate, the spin susceptibility should be Pauli-like with no obvious temperature dependence, which is contrary to what we observed. Supplementary Figure 12 shows the temperature dependence of bulk spin susceptibility (χ) for the same device presented in Supplementary Figure 4. The inverse of susceptibility ($1/\chi$) is approximately linear with temperature, which is consistent with the Curie law. These measurements were taken at a gate voltage of -1.0 V, which is already slightly beyond the "T_2 peak" (as shown in Figure 5, the "T_2 peak" usually occurs between -0.5 V and -0.8 V). Therefore, it is reasonable to conclude that the system is non-degenerate at the "T_2 peak".

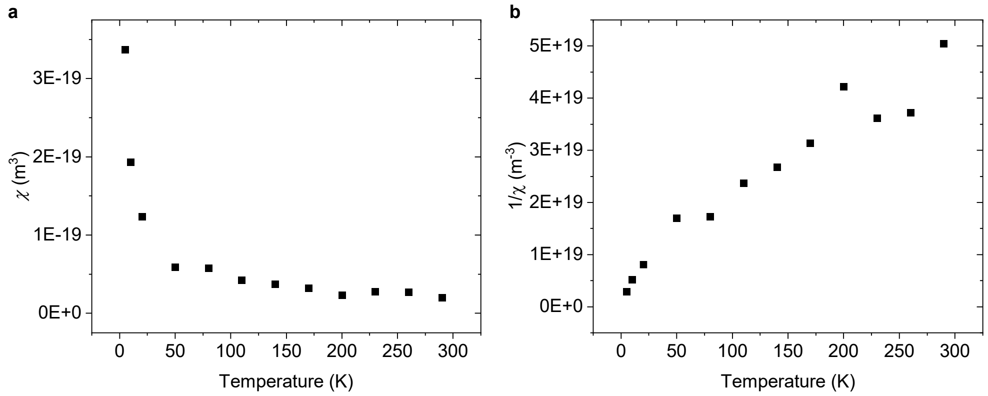

Supplementary Figure 12: **Curie-like spin susceptibility for a device at -1.0 V.** This is the same device shown in Supplementary Figure 4. The temperature dependence of χ (a) and $1/\chi$ (b) indicates that the spin susceptibility is Curie-like and the system is non-degenerate.

We also note that during our ESR measurements, there is no voltage applied between the source/drain electrodes, meaning the device behaves like a MOS capacitor. In this case, the equations in classic semiconductor device textbooks describing MOS capacitors are applicable, which are based on equilibrium statistics and crucially include the Einstein relation. Furthermore, our FI-ESR measurements were performed only after stabilization of the sample temperature—typically an hour or so—while each power saturation scan took several hours to complete. This time scale, along with the reproducibility of our results, also suggests that our devices were at equilibrium for the data reported here.

Regarding the mobility value used for the Einstein relation, it is a common practice within the rubrene transistor community that a small drain voltage should be used for transistor measurements and the mobility should be extracted from the linear regime[23] Usually in this regime, the source-drain current is Ohmic (as shown in Supplementary Figure 13), indicating the field-independent nature of carrier mobility/diffusivity. If the system were non-equilibrium, we would expect a much stronger dependence of mobility on the drain electric field, which would lead to non-Ohmic IV curves.

A more rigorous way to prove that the system is at equilibrium would be to perform a transport simulation to find the actual distribution function. However, this would require techniques such as non-equilibrium Green's function and is beyond the scope of this manuscript.

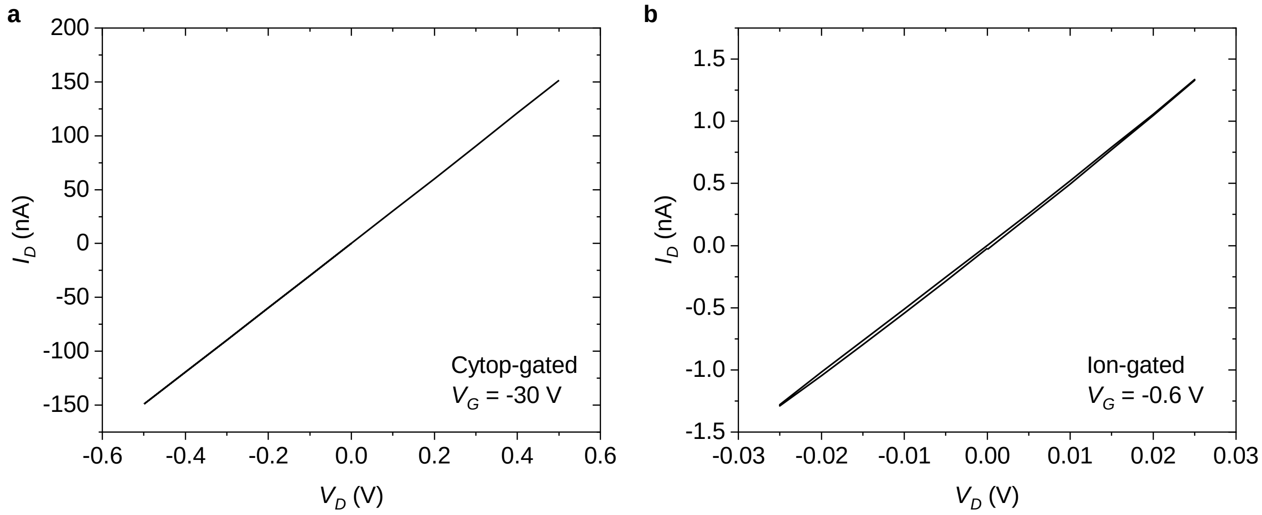

Supplementary Figure 13: **Ohmic IV curves in rubrene transistors: (a) a cytop-gated conventional FET; (b) an ion-gated EDLT.** These IV curves were measured at room temperature.

Supplementary Note 11 Difficulties in performing ion-gel-gated transistor and ESR measurements in one identical rubrene crystal

Ideally, we would like to perform in-situ ion-gel-gated transistor and ESR measurements in one identical rubrene crystal. However, the following difficulties prevented us from performing such measurements. It has been known that the rubrene/metal contact is critical for obtaining good transistor characteristics. The best rubrene transistors are fabricated using the so-called "flip-crystal" method, which creates a pristine, van der Waals rubrene/metal interface. To achieve perfect lamination on the solid substrate, the crystal needs to be thin and flexible, but usually the size of a thin crystal is not large enough. For a crystal with a sufficiently large area (like the one shown in Figure 1), it doesn't spontaneously stick to electrodes on a rigid substrate, making transistor measurements impractical. Contacts can also be made directly on top of the crystal by metal evaporation or by hand painting with Ag paint, but these methods also lead to non-ideal, contact-limited transistors with inferior performance.

Although we were unable to to perform in-situ measurements of ion-gel-gated transistor and ESR characteristics in one identical rubrene crystal, we conducted such measurements in a device based on multiple thin crystals (Supplementary Figure 7). Both the magnitude of spin lifetimes and the dependence on charge carrier density match well with the results shown in the main text. As a result, we believe our experiments on multi-crystal transistors and on one-crystal capacitors together provide a comparable level of information to what one would obtain from a one-crystal transistor device.

Supplementary References

[1] Xu, W. *et al.* Tunneling magnetoresistance observed in la0. 67sr0. 33mno3/organic molecule/co junctions. *Applied Physics Letters* **90**, 072506 (2007).

[2] Yoo, J.-W. *et al.* Tunneling vs. giant magnetoresistance in organic spin valve. *Synthetic Metals* **160**, 216–222 (2010).

[3] Lin, R., Wang, F., Rybicki, J., Wohlgenannt, M. & Hutchinson, K. Distinguishing between tunneling and injection regimes of ferromagnet/organic semiconductor/ferromagnet junctions. *Physical Review B—Condensed Matter and Materials Physics* **81**, 195214 (2010).

[4] Galbiati, M. *et al.* Is spin transport through molecules really occurring in organic spin valves? a combined magnetoresistance and inelastic electron tunnelling spectroscopy study. *Applied Physics Letters* **106**, 082408 (2015).

[5] Drogeler, M. *et al.* Spin lifetimes exceeding 12 ns in graphene nonlocal spin valve devices. *Nano Letters* **16**, 3533–3539 (2016).

[6] Lou, X. *et al.* Electrical detection of spin transport in lateral ferromagnet–semiconductor devices. *Nature Physics* **3**, 197–202 (2007).

[7] Crooker, S. *et al.* Imaging spin transport in lateral ferromagnet/semiconductor structures. *Science* **309**, 2191–2195 (2005).

[8] Ghosh, S. *et al.* Electrical control of spin coherence in zno. *Applied Physics Letters* **92**, 162109 (2008).

[9] Márkus, B. *et al.* Ultralong spin lifetime in light alkali atom doped graphene. *ACS Nano* **14**, 7492–7501 (2020).

[10] Schott, S. *et al.* Polaron spin dynamics in high-mobility polymeric semiconductors. *Nature Physics* **15**, 814–822 (2019).

[11] Yuan, H. T. *et al.* Liquid-gated electric-double-layer transistor on layered metal dichalcogenide, SnS2. *Applied Physics Letters* **98**, 012102 (2011).

[12] Xie, W., Wang, S., Zhang, X., Leighton, C. & Frisbie, C. D. High Conductance 2D Transport around the Hall Mobility Peak in Electrolyte-Gated Rubrene Crystals. *Physical Review Letters* **113**, 246602 (2014).

[13] Abragam, A. *The Principles of Nuclear Magnetism* (Oxford University Press, Amen House, London, E.C.4, 1961).

[14] Xia, Y., Xie, W., Ruden, P. P. & Frisbie, C. D. Carrier Localization on Surfaces of Organic Semiconductors Gated with Electrolytes. *Physical Review Letters* **105**, 036802 (2010).

[15] Xie, W. & Frisbie, C. D. Organic Electrical Double Layer Transistors Based on Rubrene Single Crystals: Examining Transport at High Surface Charge Densities above 10 ^{13} cm $^{-2}$. *The Journal of Physical Chemistry C* **115**, 14360–14368 (2011).

[16] Podzorov, V. *et al.* Intrinsic Charge Transport on the Surface of Organic Semiconductors. *Physical Review Letters* **93**, 086602 (2004).

[17] Podzorov, V., Menard, E., Rogers, J. A. & Gershenson, M. E. Hall Effect in the Accumulation Layers on the Surface of Organic Semiconductors. *Physical Review Letters* **95**, 226601 (2005).

[18] Hulea, I. N. *et al.* Tunable Fröhlich polarons in organic single-crystal transistors. *Nature Materials* **5**, 982–986 (2006).

[19] Xie, W. *et al.* High-mobility transistors based on single crystals of isotopically substituted rubrene-d 28. *The Journal of Physical Chemistry C* **117**, 11522–11529 (2013).

[20] Ren, X. *et al.* Negative isotope effect on field-effect hole transport in fully substituted 13c-rubrene. *Advanced Electronic Materials* **3**, 1700018 (2017).

[21] Xie, W. *et al.* Utilizing carbon nanotube electrodes to improve charge injection and transport in bis (trifluoromethyl)-dimethyl-rubrene ambipolar single crystal transistors. *ACS nano* **7**, 10245–10256 (2013).

[22] Zimmerling, T. & Batlogg, B. Improving charge injection in high-mobility rubrene crystals: From contact-limited to channel-dominated transistors. *Journal of Applied Physics* **115**, 164511 (2014).

[23] Choi, H. H., Cho, K., Frisbie, C. D., Sirringhaus, H. & Podzorov, V. Critical assessment of charge mobility extraction in fets. *Nature Materials* **17**, 2–7 (2018).

[24] Elsner, J. *et al.* Thermoelectric transport in molecular crystals driven by gradients of thermal electronic disorder (2024). `2406.18785`.

[25] Maaß, H. *et al.* Spin-texture inversion in the giant rashba semiconductor bitei. *Nature Communications* **7**, 11621 (2016).

[26] Feng, Y. *et al.* Rashba-like spin splitting along three momentum directions in trigonal layered ptbi2. *Nature Communications* **10**, 4765 (2019).

[27] Machida, S.-i. *et al.* Highest-occupied-molecular-orbital band dispersion of rubrene single crystals as observed by angle-resolved ultraviolet photoelectron spectroscopy. *Physical Review Letters* **104**, 156401 (2010).

[28] Ding, H., Reese, C., Mäkinen, A. J., Bao, Z. & Gao, Y. Band structure measurement of organic single crystal with angle-resolved photoemission. *Applied Physics Letters* **96**, 222106 (2010).

[29] Huang, Y. *et al.* Unraveling the crucial role of trace oxygen in organic semiconductors. *Nature Communications* **15**, 626 (2024).

[30] Takase, K., Ashikawa, Y., Zhang, G., Tateno, K. & Sasaki, S. Highly gate-tuneable rashba spin-orbit interaction in a gate-all-around inas nanowire metal-oxide-semiconductor field-effect transistor. *Scientific Reports* **7**, 930 (2017).

[31] Wetzelaer, G., Koster, L. & Blom, P. Validity of the einstein relation in disordered organic semiconductors. *Physical Review Letters* **107**, 066605 (2011).

[32] Troisi, A. Dynamic disorder in molecular semiconductors: Charge transport in two dimensions. *The Journal of Chemical Physics* **134**, 034702 (2011).
